# *E. coli* Sepsis: Red Flag for Colon Carcinoma—A Case Report and Review of the Literature

**DOI:** 10.1155/2017/2570524

**Published:** 2017-06-13

**Authors:** Hiren G. Patel, Seme Tabassum, Sohail Shaikh

**Affiliations:** ^1^Department of Gastroenterology & Hepatology, New York Medical College/St. Joseph's Regional Medical Center, Paterson, NJ, USA; ^2^Department of Internal Medicine, New York Medical College/St. Joseph's Regional Medical Center, Paterson, NJ, USA

## Abstract

We present an atypical case of newly diagnosed colon cancer and provide insight into the infectious predispositions of* E. coli* bacteremia to the development of colon adenocarcinoma. A 66-year-old female was admitted to the hospital with one-week symptoms of subjective fevers, chills, and lower back pain. Upon initial evaluation, her temperature was 101 degrees Fahrenheit with a white count of 12,000 K/mm^3^. Initial septic workup was positive for* E. coli* bacteremia. The patient was started on Aztreonam. Repeat blood culture 48 hours later was negative for any growth. However, later during hospital stay blood culture was repeated due to SIRS, which was positive again for* E. coli*. CT scan of the chest/abdomen/pelvis with contrast revealed no signs of colitis. Without clear etiology for recurrent* E. coli* bacteremia ultimately colonoscopy was performed which showed an ulcerated mass in the cecum. Biopsy showed moderately differentiated adenocarcinoma.* E. coli* strains B2 and D produce cyclomodulin toxins as part of their virulence, which interferes with the cell cycle regulation, promoting chromosomal instability, and increasing susceptibility to cancer. In patients with recurrent* E. coli* bacteremia with an unknown source, colonoscopy should be done to look for colon cancer.

## 1. Introduction

Colorectal cancer (CRC) is the third most commonly diagnosed cancer worldwide with an estimated 135,430 new cases in the United states alone in 2017 [1]. While the relationship between* Streptococcus bovis* bacteremia and colon cancer has been well established, rare cases of colon adenocarcinoma have been reported after exposure to other microorganisms. We present an atypical case of newly diagnosed colon cancer and provide insight into the infectious predispositions of* E. coli* bacteremia to the development of colon adenocarcinoma.

## 2. Case Report

A 66-year-old female with past medical history of hypertension, type 2 diabetes mellitus, and obesity was evaluated for one-week symptoms of subjective fevers, chills, and lower back pain. Upon initial evaluation, she exhibited pyrexia (101 degrees Fahrenheit) with a leukocytosis of 12,000 K/mm^3^. She denied cough, chest pain, shortness of breath, fatigue, weight loss, hemoptysis, recent travel, or sick contacts. Chest X-ray on admission showed bibasilar infiltrates and she was admitted for community-acquired pneumonia. Her procalcitonin level on admission was 29.02 K/mm^3^, lactic acid of 4.8 MMOL/L. Initial septic workup was positive for* E. coli* bacteremia. The patient was started on Aztreonam (due to her penicillin allergy) and Levaquin. Repeat blood culture 48 hours later was negative for any growth. However, blood culture was repeated due to SIRS, which were positive again for* E. coli*. The patient reported no abdominal complaints other than chronic constipation. Of note, the patient had cholecystectomy many years ago. On admission, her hemoglobin was 11.0 G/DL (baseline unknown), hematocrit was 35.1%, MCV was 82.8 u^3^, and the platelet count was 106 K/mm^3^. The fecal occult blood test was negative. The patient has no history of anemia, illicit drug use, and family history of colorectal cancer and never had a previous colonoscopy or endoscopy. CT scan of the chest/abdomen/pelvis with contrast revealed no signs of colitis; however, it was remarkable for spondylitis/diskitis and adjacent fluid collection at L4-L5. The patient underwent a CT-guided aspiration biopsy of the L4-L5 discitis and adjacent fluid collection, showing no growth on the culture. Without clear etiology for recurrent* E. coli* bacteremia ultimately colonoscopy was performed which showed an ulcerated cecal mass (2.6 × 1.8 × 0.5 cm). Biopsy showed moderately differentiated colon adenocarcinoma. MRI of the abdomen/pelvis with and without contrast showed no evidence of metastasis. The patient underwent a right hemicolectomy, after which the tumor was staged T3, N0.

## 3. Discussion

CRC presenting as persistent* E. coli* bacteremia is rare. This is the sixth case reported of CRC with preceding* E. coli* bacteremia. All previous cases of* E. coli* bacteremia preceding CRC diagnosis were presented with different nonspecific complaints like right leg pain, intermittent fever, fatigue, lower back pain, and weight loss [2–6].

Molecular research has supported the relationship of CRC in patients with preceding bacterial infections.* Streptococcus bovis* proteins have been suggested to overexpress the COX-2 genes, increasing colorectal cancer by inhibiting apoptosis and increasing angiogenesis [7]. Pathogenic* E. coli* strains also are thought to play a role in predisposing patients to colorectal cancer. Although* Escherichia coli* is a commensal inhabitant of the human colon from birth, there are several factors that may lead to intestinal dysbiosis, making enterocytes more vulnerable to the development of adenocarcinoma [8]. The* E. coli* phylogenic groups including A, B1, B2, D, and E are classified based on their virulence factors among which strains B2 and D have been found to be the pathogenic strains as they release cyclomodulin toxins as part of their virulence, thus interfering with cell cycle regulation, promoting chromosomal instability, and increasing susceptibility to cancer [8]. An increased prevalence of cyclomodulin-producing B2 strain of* E. coli* was seen in stage III/IV colon cancer compared to those with stage I colon cancer, suggesting a correlation between the existence of* E. coli* with the development and prognosis of colon cancer [9]. Multiple studies show that pathogenic* E. coli* strains have the potential to transform enterocytes genetically by their cyclomodulin toxin effects and promote the development of colorectal cancer [8, 10]. Seven virulence factors promoting mucosal adherence and mucosal translocation were found to be encoded by the genes, papC, sfaD/E, cnf1, usp, agn43, hlyA, and iutA, and these factors were, again, predominant in the B2 pathogenic strains of* E. coli* [11]. The virulence of particularly the B2 subtype of* E. coli*, within the carcinomatous cells, could potentially cause the bowel to blood translocation, thus causing* E. coli* bacteremia.

Colonic mucosal* E. coli* isolates from CRC and ulcerative colitis more commonly express polyketide synthase gene complex (pks) pathogenicity whose gene products cause epithelial DNA damage and induce tumors in inflammation associated CRC [12]. afa-1 gene which induces growth factors that lead to angiogenesis and carcinogenesis was also found higher among colonic mucosal* E. coli* of inflammatory bowel disease and colorectal cancer cells [12]. Enteropathogenic* E. coli* was reported to downregulate expression of key DNA mismatch repair proteins MSH2 and MLH1 [13]. Jin et al. reported that that hemolytic* E. coli* significantly induced colonic tumorigenesis in female mice by activating the oncogenic protein GLUT1 expression and repressing the tumor suppressor BIM expression by elevating levels of HIF-1*α*, in a manner dependent on the hly-encoded alpha hemolysin [14]. Jin et al. also reported that as hly^+^ type 1,* E. coli* is associated with adenoma and CRC can be used as potential antecedent biomarker of CRC [14].

Previous studies have concluded that fever of unknown origin should prompt a search for a hidden malignancy. Considering our case and the ones recaptured in this report, we propose that a colonoscopy should be considered for patients with recurrent* E. coli* bacteremia with an unknown source, particularly when imaging studies are unremarkable.
